# *Serpula* and *Spiraserpula* (Polychaeta, Serpulidae) from the Tropical Western Atlantic and Gulf of Guinea

**DOI:** 10.3897/zookeys.198.3030

**Published:** 2012-05-30

**Authors:** Rolando Bastida-Zavala

**Affiliations:** 1 Laboratorio de Sistemática de Invertebrados Marinos (LABSIM), Universidad del Mar, campus Puerto Ángel, Ciudad Universitaria, Apdo. Postal 47, Puerto Ángel, Oaxaca, 70902, México

**Keywords:** Annelida, Bahamas, Caribbean, new records, new species, taxonomy, Turks and Caicos

## Abstract

Six species of *Serpula* and *Spiraserpula* were identified, mainly, from the material of the expeditions of the Rosenstiel School of Marine and Atmospheric Science, University of Miami, including two new species of *Serpula*. *Serpula madrigalae* **sp. n.** from the Turks and Caicos has a tube with five longitudinal ridges, four rows of alveoli and a medium-sized shallow symmetrical opercular funnel with 17 radii, and an inner surface with opercular tubercles. *Serpula vossae* **sp. n.** from the Western Caribbean and Bahamas has a tube with 6–8 longitudinal ridges, and a large, deep symmetrical opercular funnel, with 21–33 radii, and a smooth inner surface. *Serpula* cf. *vermicularis*, recorded from the Gulf of Guinea (tropical eastern Atlantic), is distinguished from the nominal species in possessing fewer opercular radii (33–39) and the lack of a proximal rasp in the bayonet chaetae; tubes are missing. The distribution range is extended for the three known *Spiraserpula* species found in the collections, *Spiraserpula caribensis*, *Spiraserpula karpatensis* and *Spiraserpula ypsilon*.

## Abstract (es)

Seis especies de *Serpula* y *Spiraserpula* fueron identificadas, principalmente del material de las expediciones de la Rosenstiel School of Marine and Atmospheric Science, University of Miami, incluyendo dos nuevas especies de *Serpula*. *Serpula madrigalae* **sp. n.** es descrita de las Turks y Caicos, se caracteriza por tener un tubo con cinco costillas longitudinales, con cuatro hileras de alvéolos y un embudo simétrico, mediano y somero, con 17 radios, y la superficie opercular interna con tubérculos. *Serpula vossae* **sp. n.** es descrita del Caribe occidental y Bahamas; su tubo tiene 6–8 costillas longitudinales, un embudo opercular simétrico, largo y profundo, con 21–33 radios, y la superficie opercular interna lisa. *Serpula* cf. *vermicularis* es registrada del golfo de Guinea (Atlántico oriental tropical); se distingue de la especie nominal por tener menos radios operculares (33–39) y le falta la denticulación fina proximal en las setas bayoneta, los tubos se perdieron. Para las tres especies de *Spiraserpula* halladas, *Spiraserpula caribensis*, *Spiraserpula karpatensis* y *Spiraserpula ypsilon*, se amplió el ámbito de distribución.

## Introduction

*Serpula* Linnaeus, 1758 the type genus of the polychaete family Serpulidae Rafinesque, 1815, has 31 species (ten Hove and Kupriyanova 2009, Pillai 2009). Six species have been described in the Eastern Atlantic and Mediterranean, including the type species, *Serpula vermicularis* Linnaeus, 1767, *Serpula concharum* Langerhans, 1880, *Serpula lobiancoi* Rioja, 1917, *Serpula planorbis* Southward, 1963, *Serpula israelitica* Amoureux, 1976 and *Serpula cavernicola* Fassari & Mòllica, 1991. However, there are taxonomic problems in some species because they were poorly described and/or recorded from widely separated localities. For example, *Serpula vermicularis* has been recorded from several tropical, subtropical, temperate and cold water localities of the world (Kupriyanova 1999). There is a consensus now that *Serpula vermicularis*, previously considered to be a cosmopolitan species, is ill-defined and its distribution is possibly restricted to temperate and cold waters of the North Atlantic Ocean and Mediterranean (ten Hove and Jansen-Jacobs 1984, Imajima and ten Hove 1984, Kupriyanova and Jirkov 1997, Kupriyanova 1999, ten Hove and Kupriyanova 2009).

In the Western Atlantic, the genus *Serpula* is very poorly known, as only two species have been recorded: *Serpula vermicularis granulosa* by Day (1973) from Beaufort, North Carolina, and *Serpula* sp. A by ten Hove and Wolf (1984) from the northeastern Gulf of Mexico. Another taxon, *Serpula sombreriana* McIntosh, 1885, from Sombrero and St. Thomas Islands, lacks an operculum and was therefore transferred to *Hyalopomatus* (Ben-Eliahu and Fiege 1996).

The current state of our knowledge on *Serpula* species, compared to that of almost 100 species of *Hydroides* (ten Hove and Kupriyanova 2009, Pillai 2009), is probably explained by the fact that most tropical *Serpula* species are sublittoral and many extend their distribution into deeper waters. In cold and temperate waters, *Serpula* species can be present in shallow waters, attain larger sizes (Kupriyanova 1999), and form large aggregations, even reefs (Ramos and San Martin 1999).

*Spiraserpula* Regenhardt, 1961 was established initially for fossil serpulids. Pillai and ten Hove (1994) revised the Recent species belonging to the genus. They described 16 species out of 19 currently included in the genus, and eight of them were from the Caribbean. The main distinguishing feature of this genus is their complex internal tube structures (ITS), described by Pillai and ten Hove (1994). Unfortunately, the species remain mostly unknown to non-specialists, mainly because they are tiny, most are sublittoral, and often overlooked or confused with other taxa.

This work is part of a larger study examining subtidal and deep sea serpulids from the Grand Caribbean region and from the Gulf of Guinea, tropical eastern Atlantic.

## Materials and methods

Between 1963 and 1975, the Rosenstiel School of Marine and Atmospheric Science (RSMAS) conducted the University of Miami Deep Sea Expeditions aboard of R/V Gerda, John Elliot Pillsbury, James M. Gillis and Columbus Iselin, and sampled more than 3,350 stations from the Gulf of Panama, throughout the Caribbean to the Gulf of Guinea, the Straits of Florida, the Bahamas, the area northward to the Bermudas and the deep basins and the deep waters, from the intertidal to 8,650 m in the Puerto Rico Trench (Voss et al. 1977, Bastida-Zavala et al. 2001). The revision of the serpulid material from these expeditions resulted in finding of 11 *Serpula* and 10 *Spiraserpula* specimens from the western Caribbean, Bahamas, Turks and Caicos, Los Roques Islands, Trinidad and Tobago, and in the Gulf of Guinea.

Additionally, two specimens of *Serpula* (recorded by Bastida-Zavala and Salazar-Vallejo 2000) and 14 specimens of *Spiraserpula* of the collections of El Colegio de la Frontera Sur and the Instituto de Oceanología of Cuba were available for study. Type specimens were deposited in the National Museum of Natural History, Smithsonian Institution, Washington, D.C. Other specimens were deposited in the collections of the respective lending institutions.

The specimens of *Serpula* and *Spiraserpula* were fixed with 10% formalin and preserved with 70% alcohol. They were studied in a standardized way (ten Hove and Jansen-Jacobs 1984, Bastida-Zavala and ten Hove 2002). Line drawings were made using a camera lucida, and the photographs were taken with a digital camera Canon G11 fitted to a microscope adapter.

The main standard measurements and observations on *Serpula* were: total length (measured from most distal part of the operculum to the pygidium), thoracic width (measured from the collar region level), number of thoracic chaetigers, number of radioles in each lobe of the branchial crown, number of longitudinal ridges on the tube (not counting basal ridges attached to the substratum), presence or absence of peristomes, transverse ridges or alveoli on the tube, opercular length (measured from the base of funnel, or constriction, if present, to the tips of the radii), opercular diameter (measured across the distal part of the funnel), number of funnel radii, number of teeth on bayonet chaetae and the presence or absence of a proximal rasp in these chaetae. An exploratory analysis of the number of opercular radii and body length ratio of the *Serpula* species is included. Scales of figures and photographs are in millimeters.

### The following abbreviations are used in the text:

**Collections**

**ECOSUR** Colección de Referencia. El Colegio de la Frontera Sur, Chetumal, Quintana Roo, México.

**UMML** Marine Invertebrate Museum, Rosenstiel School of Marine and Atmospheric Science, University of Miami, Miami, Florida, USA.

**UMAR** Colección de Invertebrados Marinos, Universidad del Mar, Puerto Ángel, Oaxaca, México.

**USNM** National Museum of Natural History, Washington D.C., USA.

**Characters**

**OL** Opercular length

**OD** Opercular diameter

**THW** Thoracic width

**TL** Total length of the body

**Statistical terms**

n sample size

r: range of data

µ mean

± standard deviation

## Systematics

**Class Polychaeta Grube, 1850**

**Family Serpulidae Rafinesque, 1815**

### Genus *Serpula* Linnaeus, 1758

**Type species.** ***Serpula vermicularis* Linnaeus, 1767** by subsequent designation (Heppell 1963) under the plenary powers of the International Commission on Zoological Nomenclature (Evans and China 1966).

#### Serpula madrigalae sp. n.

urn:lsid:zoobank.org:act:1EACCEA3-111D-4F4A-AF5B-CD03E15FBF6C

http://species-id.net/wiki/Serpula_madrigalae

Figs 1A–D 2A–G 5 6

##### Type locality.

**Turks and Caicos.** East of Caicos Island.

##### Type material.

**Turks and Caicos.** Holotype (USNM 1157006), RV Pillsbury, cruise 7106, sta. 1423, 21°41'N, 71°23'W, 10-feet otter trawl, 18 m, July 19, 1971 (ex UMML 22.1054).

##### Description.

**Figure 1.**
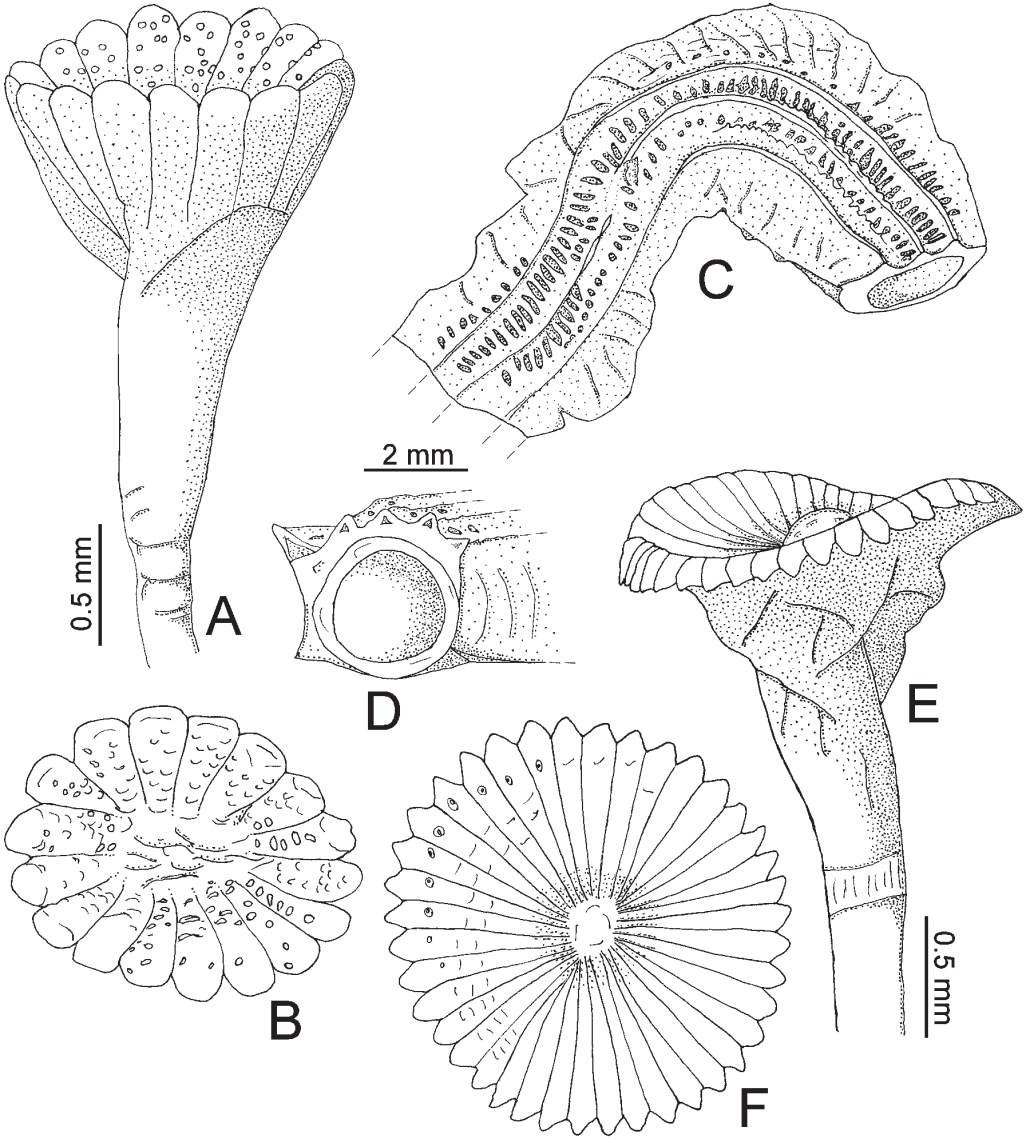
**A–D** *Serpula madrigalae* sp. n., from Turks and Caicos Islands, USNM 1157006, holotype **A–B** operculum in lateral and aboral views **C–D** tube in dorsal and frontal views **E–F** *Serpula* cf. *vermicularis*, from Nigeria, UMML 22.545 **E–F** operculum in lateral and aboral views.

**Figure 2.**
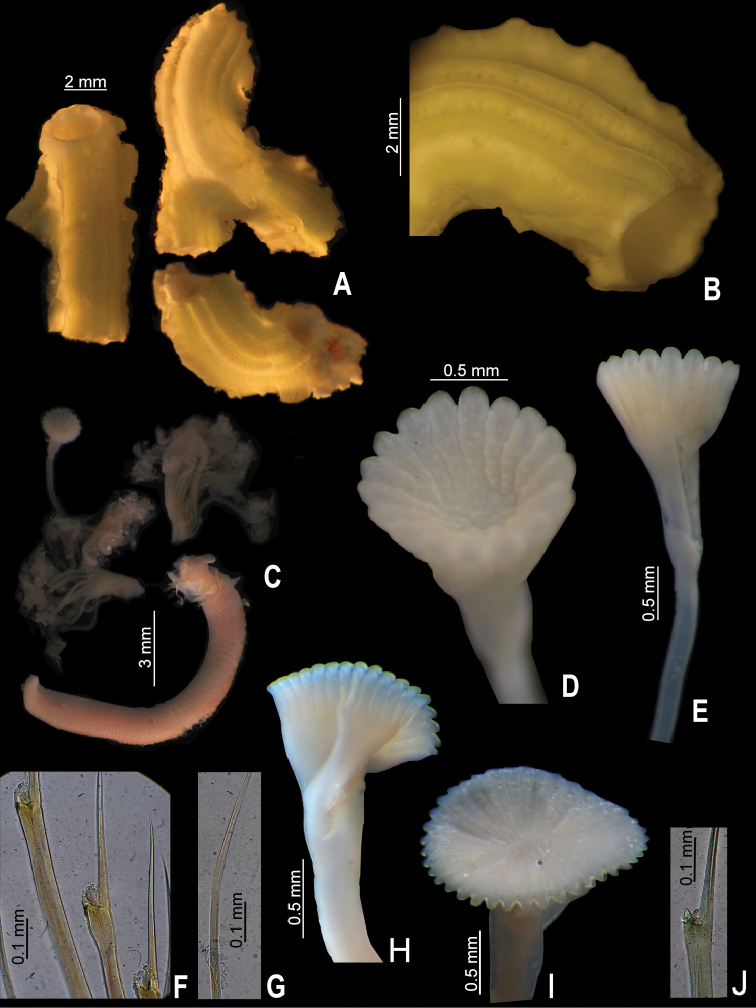
**A–G** *Serpula madrigalae* sp. n., from Turks and Caicos, USNM 1157006, holotype **A–B** tube and detail **C** entire body **D–E** operculum, in aboral and lateral views **F** bayonet chaetae **G** hooded (capillary) chaetae **H–J** *Serpula* cf. *vermicularis*, from Nigeria, UMML 22.545 **H–I** two distinct opercula in lateral and aboral views **J** bayonet chaetae.

Tube color greenish yellow (Fig. 2A–B); with five longitudinal ridges, lateral-most ridges larger than middle ones (Figs 1C–D, 2A–B); lacking transverse ridges and peristomes; with four rows of alveoli, more evident between dorsal-most longitudinal ridges (Figs 1C, 2A–B).

Body yellowish-brown, branchial crown and operculum yellow pale (preserved material only, Fig. 2C). TL= 20 mm; THW= 1.6 mm. Branchial crown with 18 radioles in each lobe; lacking branchial membrane.

Peduncle smooth, with well-defined constriction (Fig. 2D); inserted in left lobe. Club-shaped pseudoperculum present.

Operculum with moderately long, shallow, symmetrical funnel; lacking bulbous basal part (Figs 1A, 2D–E). OL= 2.3 mm, OD= 1.4 mm. Interradial grooves 1/3 of funnel length (Figs 1A, 2E). Funnel has 17 radii with rounded tips. Opercular inner surface with irregular tubercles (Figs 1B, 2D).

Collar thick, with short ventral and dorsal lobes. Thorax consists of seven chaetigers. Collar chaetal fascicles symmetrical with regard to size and composition, unlike in some specimens of *Serpula vossae* sp. n. Bayonet chaetae with two blunt-elongate teeth, distal blade smooth, lacking proximal rasp (Figs 2F); hooded (capillary) chaetae present (Fig. 2G).

Thoracic membranes well developed, narrowing toward to last thoracic chaetigers, fused ventrally, forming a short apron. Remaining six thoracic chaetigers with hooded (limbate) chaetae of two sizes; saw-shaped uncini.

Anterior part of abdomen lacking distinct achaetous region. Anterior and middle abdominal chaetigers with flat-trumpet chaetae. Posterior chaetigers with ‘capillary’ chaetae. Anterior and posterior uncini saw-shaped.

##### Etymology.

Named after my wife, Dr Socorro García-Madrigal, a specialist on crustaceans, who gave me the necessary encouragement and time to undertake this research.

##### Distribution.

Only recorded from the vicinity of Caicos Island, Turks and Caicos Islands (Fig. 6).

##### Ecology.

Sublittoral, 18 m. In the same sample there were other serpulids: *Pomatostegus stellatus*, *Pseudovermilia multispinosa*, *Spirobranchus giganteus*, and *Vermiliopsis annulituba*.

##### Remarks.

*Serpula madrigalae* sp. n. resembles other *Serpula* species with symmetrical,moderately long and shallow funnels, such as *Serpula cavernicola*, *Serpula granulosa* Marenzeller, 1884, *Serpula israelitica* Amoureux, 1976, *Serpula jukesii* Baird, 1865, *Serpula narconensis* Baird, 1865, *Serpula oshimae* Imajima & ten Hove, 1984, *Serpula tetratropia* Imajima & ten Hove, 1984, *Serpula vermicularis* Linnaeus, 1767, and *Serpula zelandica* Baird, 1865. However, *Serpula madrigalae* sp. n. differs from all other *Serpula* species with regard to its characteristic tube which has five longitudinal ridges and four rows of alveoli (Figs 1C–D, 2A–B).

*Serpula madrigalae* sp. n. resembles *Serpula vermicularis granulosa*, in having tubercles on the internal surface of the operculum; however, the diagnosis of the latter species was brief (Day 1973). At least *Serpula madrigalae* sp. n. differs by the tube with five longitudinal ridges and four rows of alveoli (Figs 1C–D, 2A–B), while *Serpula vermicularis granulosa* is “faintly ridged” (Day 1973:131); also, Day (1973) mentioned more opercular radii (20–40) than present in *Serpula madrigalae* sp. n. (17, Figs 1B, 5).

*Serpula madrigalae* sp. n. also resembles *Serpula* sp. A, from the northeastern part of the Gulf of Mexico, with regard to the shape of the operculum, the number of radii and the depths from which they were collected. However, they differ with regards to other features: *Serpula madrigalae* sp. n. has irregular tubercles on the internal surface of the operculum (Figs 1B, 2D) and lacks a proximal rasp in the bayonet chaetae (Fig. 2F), while *Serpula* sp. A lacks tubercles (ten Hove and Wolf 1984, Fig. 55–8a) and has bayonet chaetae with a proximal rasp. Additionally, ten Hove and Wolf (1984) mentioned that all the specimens lacked their tubes. Hence is not possible to assign the specimens recorded as *Serpula* sp. A. to *Serpula madrigalae* sp. n.

#### Serpula vossae sp. n.

urn:lsid:zoobank.org:act:3165E4EF-A4B8-47B2-B500-D5C8C1557646

http://species-id.net/wiki/Serpula_vossae

Figs 3A–D 4A–J 5 6

Serpula sp. Bastida-Zavala and Salazar-Vallejo, 2000:852–854, fig. 4B–K.

##### Type locality.

**Honduras.** Southwest of Honduras.

##### Type material.

Holotype (USNM 1157004), RV Pillsbury, cruise 6802, sta. 629, 15°58'N, 86°09'W, 40 m, March 21, 1968 (ex UMML 22.611); paratype (USNM 1157005), RV Pillsbury, cruise 6802, sta. 628, Honduras, East of Cayos Cochinos, 15°57'N, 86°15'W, 47 m, March 21, 1968 (ex UMML 22.610).

##### Additional material.

**Guatemala.** One complete specimen (UMML 22.1053) RV Pillsbury, cruise 6802, sta. 613, West of Punta Cortes, 15°58'N, 88°20'W, 10-feet otter trawl, 39 m, March 19, 1968. **México.** One complete specimen (ECOSUR s.n.) RV Edwin Link sta. 2792, 13 km from East of Isla Mujeres, Quintana Roo, 21°14'N, 86°36'W, 130 m, August 28, 1990, E. Escobar and L. Soto leg. **Cuba.** One complete specimen (Instituto de Oceanología de Cuba) Cayo Diego Pérez, Golfo de Batabanó, 15 m, July 20, 1988, D. Ibarzábal leg. **Bahamas.** Two complete specimens (UMML 22.435) RV Gerda, cruise 6433, sta. 391, North of Bahamas, 27°20'N, 79°11'W, screen dredge, 68 m, September 19, 1964.

##### Description.

Tube color brownish, or light brown to white; with 6–8 longitudinal ridges, all similar in size; some tubes with shallow transverse ridges, forming a rugged surface, other tubes lacking transverse ridges; most tubes lacking peristomes, two have only one peristome with appearance of a groove with shallow growth lines. Tubes lacking alveoli (Fig. 4A, C–D).

Body pale yellow (preserved material only, Fig. 4B). TL= 38.5 mm (n=7, r:20–45.5, µ=36.6 ±10.3); THW= 2 mm (n=7, r:1.5–3.4, µ=2.3 ±0.6). Branchial crown with 29 radioles (n=7, r:19–37, µ=30.9 ±6.4) left, and 29 right (n=7, r:12–35, µ=30.9 ±8.8); lacking branchial membrane (Fig. 4E).

**Figure 3.**
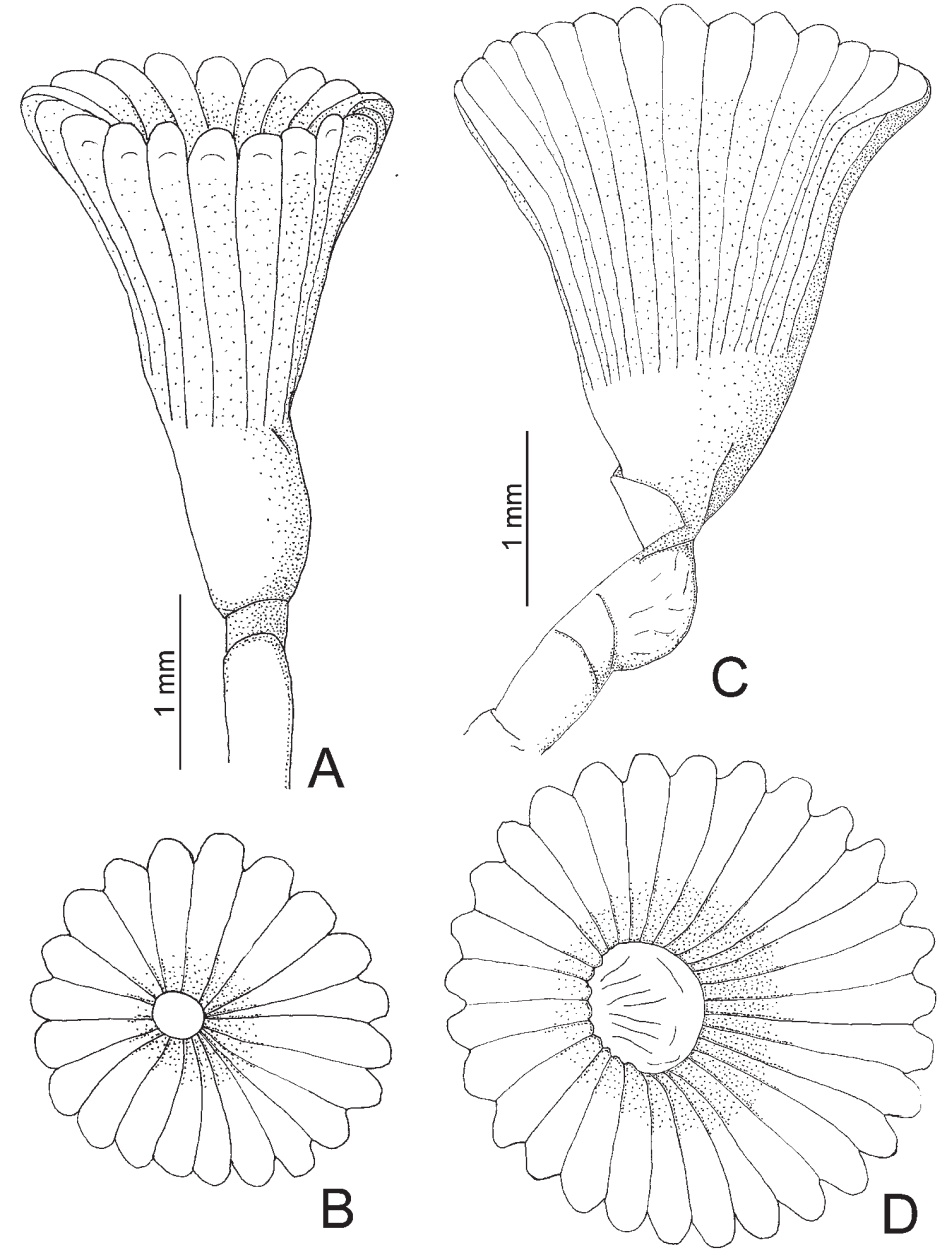
**A–D** *Serpula vossae* sp. n., from Honduras, USNM 1157004, holotype **A–B** operculum in lateral and aboral views; from Bahamas, UMML 22.435 **C–D** operculum in lateral and aboral views.

**Figure 4.**
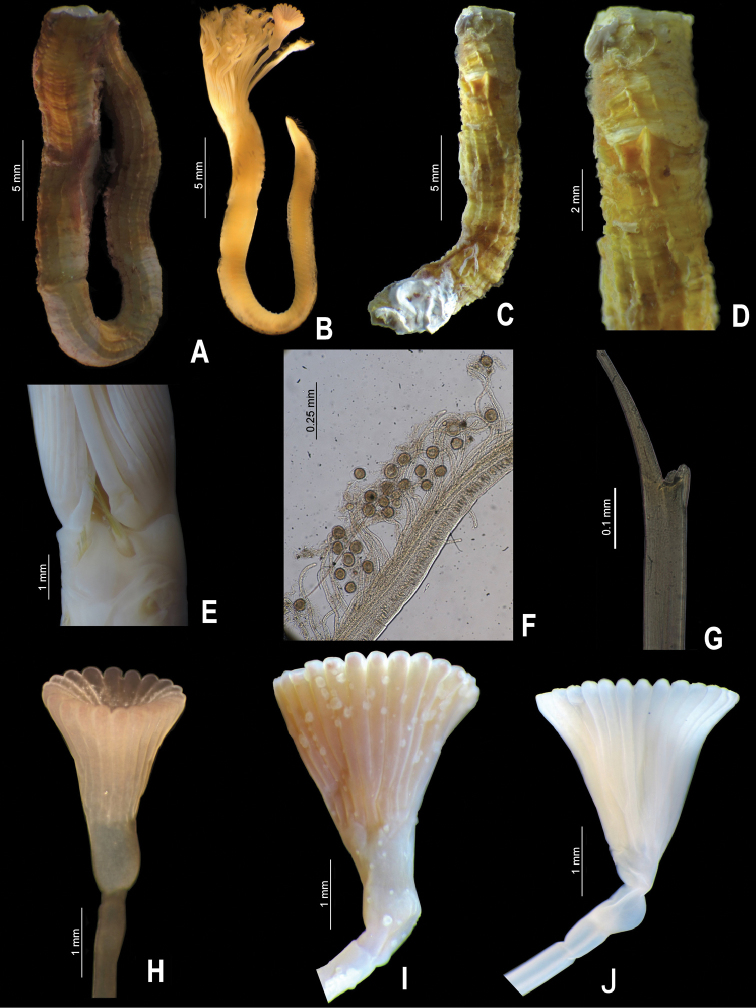
**A–J** *Serpula vossae* sp. n., from Honduras, USNM 1157004, holotype **A** tube **B** entire body; from Guatemala, UMML 22.1053 **C–D** tube and detail of peristome; from Bahamas, UMML 22.435 **E** collar region; from Cuba, IO **F** radiole with eggs; from Honduras, USNM 1157004, holotype **G**  bayonet chaetae **H** operculum; from Guatemala, UMML 22.1053 **I** operculum; from Bahamas, UMML 22.435 **J** operculum.

Peduncle smooth with insertion on left (n=2) or right (n=5); with shallow (n= 4) to well-defined constriction (n= 3) (Figs 3A, C, 4H–J). Pseudoperculum club-shaped, present in all specimens.

Operculum with long, deep symmetrical funnel; with a slightly bulbous basal part above constriction (Figs 3A, C, 4H–J). OL= 3.2 mm (n=7, r:2–4.5, µ=3.3 ±0.8), OD= 2 mm (n=7, r:1.4–2.8, µ=2.3 ±0.5). Interradial grooves 2/3 of funnel length. Funnel with 21 radii (n=7, r:21–33, µ=27.4 ±3.7) with rounded tips (Figs 3A, C, 4H–J). Opercular inner surface lacking tubercles (Fig. 3A–D).

Collar thick, with short ventral and dorsal lobes. Thorax consists of seven chaetigers. Collar fascicles in three specimens asymmetrical with regard to sizes and number of chaetae; right fascicle with larger and more chaetae than left fascicle (Fig. 4E). Bayonet chaetae with two blunt-elongate teeth, distal blade smooth, lacking proximal rasp (Fig. 4G); hooded (capillary) chaetae present.

Thoracic membranes well developed, narrowing toward last thoracic chaetigers, fused ventrally, forming a short apron. Remaining six thoracic chaetigers with hooded (limbate) chaetae of two sizes; saw-shaped uncini.

Anterior part of abdomen lacks a distinct achaetous region. Anterior and middle abdominal chaetigers with flat-trumpet chaetae. Posterior chaetigers with ‘capillary’ chaetae. Anterior and posterior uncini saw-shaped.

##### Variation.

Operculum of holotype (USNM 1157004) has roseate radial tips (Fig. 4H); the rest of specimens are yellow to white (Fig. 4I–J). Operculum and radioles of specimen from Guatemala (UMML 22.1053) have hard particles adhered, possibly salt concretions (Fig. 4I); operculum more rigid compared with the other specimens.

##### Etymology.

Named after Professor Nancy Voss, a distinguished cephalopod specialist and Director of the Marine Invertebrate Museum, who generously loaned the serpulid samples from the oceanographic expeditions of the University of Miami.

##### Distribution.

Tropical Caribbean. Bahamas, Cuba, Mexican Caribbean, Guatemala and Honduran Caribbean (Fig. 6).

##### Ecology.

Sublittoral, 15 to 130 m. On rocky and sandy bottoms, and associated with siliceous sponges and several syllid polychaetes specimens. In the same samples, there were other serpulids: *Hyalopomatus* sp., *Hydroides parvus*, *Pomatostegus stellatus*, *Pseudovermilia fuscostriata*, *Pseudovermilia occidentalis*, *Spiraserpula ypsilon*, and a vermetid shell.

##### Reproductive characters.

The specimen from Cayo Diego Pérez, Cuba, has eggs adhering to the pinnules of the radioles. The eggs, circular to slightly oval, are 55–68 µm (Fig. 4F).

##### Remarks.

**Figure 5.**
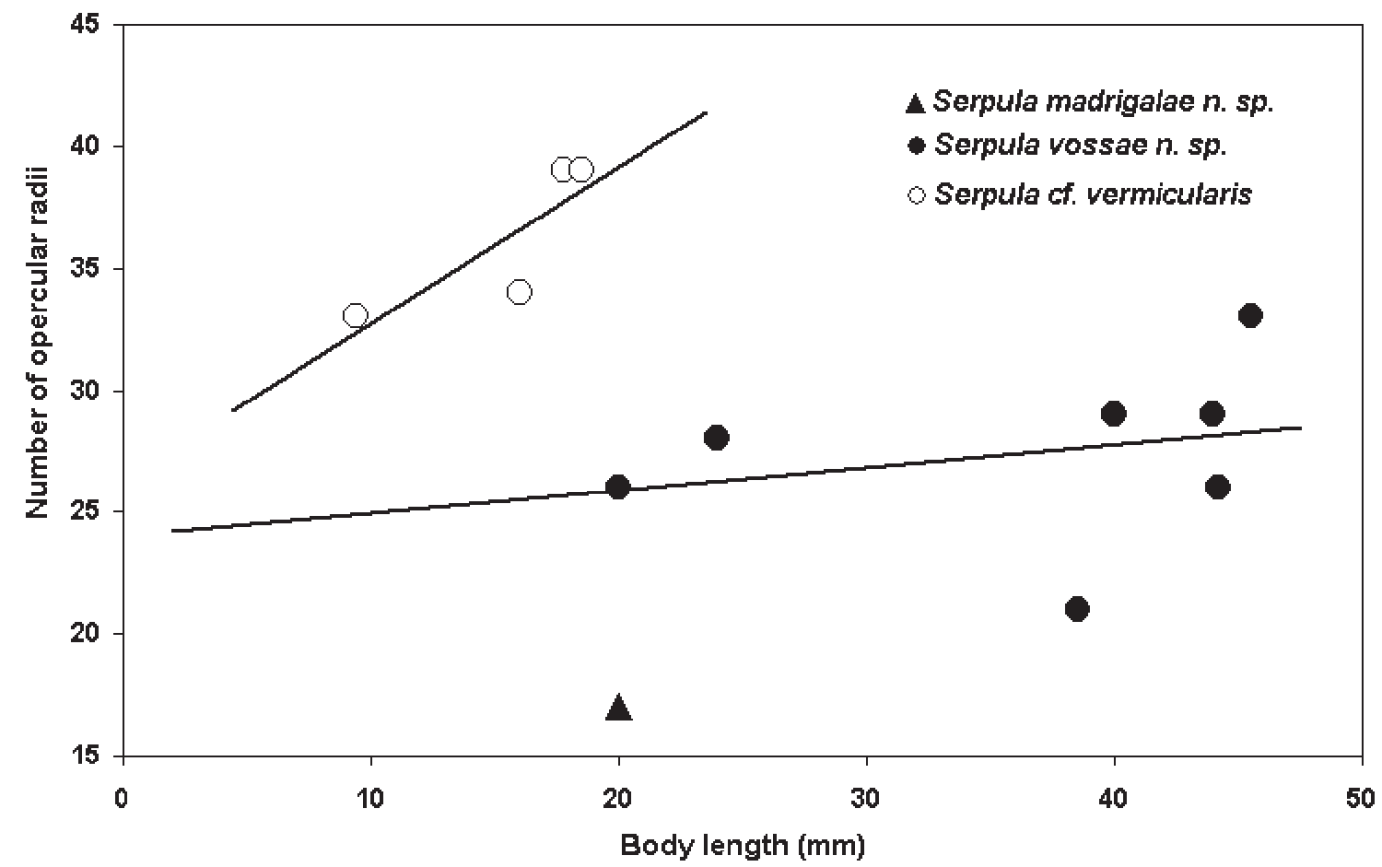
Exploratory analysis of the number of opercular radii and body length ratio: *Serpula madrigalae* sp. n. (n= 1, only for reference), *Serpula vossae* sp. n. (n= 7), and *Serpula* cf. *vermicularis* (n= 4).

*Serpula vossae* sp. n. resembles other *Serpula* species with long and deep symmetrical funnels, as in *Serpula columbiana* Johnson, 1901, *Serpula concharum* Langerhans, 1880, *Serpula longituba* Imajima, 1979, *Serpula sinica* Wu & Chen, 1979, *Serpula uschakovi* Kupriyanova, 1999, *Serpula vittata* Augener, 1914, and *Serpula watsoni* Willey, 1905. However, *Serpula vossae* sp. n. differs in having an operculum with a smooth inner surface, while *Serpula watsoni* has tubercles(Pillai 2009); *Serpula vossae* sp. n. has fewer opercular radii (21–33, Figs 4B–D, 5) than *Serpula columbiana* (55–160) or *Serpula uschakovi* (62–136) (Kupriyanova 1999); *Serpula vossae* sp. n. has 6–8 longitudinal ridges in the tube (Fig. 4A, C–D), while *Serpula columbiana*, *Serpula longituba* and *Serpula uschakovi* lack longitudinal ridges (Imajima 1979, Kupriyanova 1999), whereas *Serpula concharum*, *Serpula vittata*, and *Serpula watsoni* have five or less (Rioja 1931, Imajima 1977, 1982); *Serpula vossae* sp. n. has more radioles per branchial lobe (19–37) than *Serpula concharum* (6–15), *Serpula longituba* (9–10), *Serpula sinica* (13), while *Serpula uschakovi* has even more (43–61) than *Serpula vossae* sp. n. *Serpula vossae* sp. n. has collar chaetae with two teeth (Fig. 4G), while *Serpula longituba* lacks bayonet chaetae (Imajima 1979), *Serpula sinica* has bayonet chaetae lacking basal teeth (Wu et al. 1979), and *Serpula vittata* and *Serpula watsoni* have 10 and five basal teeth in the bayonet chaetae, respectively (Imajima 1977, 1982). These characters and others have been compared in Table 1.

**Table 1.** Comparison between *Serpula vossae* sp. n. and other similar species (AD) = according to published illustrations.

**Species:**	***Serpula columbiana***	***Serpula concharum***	***Serpula longituba***	***Serpula sinica***	***Serpula uschakovi***	***Serpula vittata***	***Serpula watsoni***	***Serpula vossae***
Localities	Puget Sound	Atlantic of Spain and Mediterranean	Kushimoto Harbour, South Japan	South China Sea	Sea of Japan	Micronesia, Melanesia, West Australia	Sri Lanka, Japan, Micronesia, Melanesia, South China Sea, Australia	West Caribbean and Bahamas
References	Kupriyanova 1999, Bastida-Zavala 2008	Rioja 1931, Zibrowius 1968, Bianchi 1981	Imajima 1979 (as *Semiserpula*)	Wu et al. 1979	Kupriyanova 1999	Augener 1914, Imajima 1982, Imajima and ten Hove 1984, 1986	Willey 1905, Imajima 1977, 1982, Imajima and ten Hove 1984, 1986, Pillai 2009	This work
Depth (m)	15–60	0–500	30–40	202–219	15	7–11	shallow	15–130
Tube color	white	white	white	violet-red	white	brownish	white	white to brownish
Longitudinal ridges	absent	3–5, smooth	absent	?	?	5	5	6–8
Transverse ridges	present	absent (AD)	absent	?	?	present	?	present
Peristomes	absent	absent (AD)	absent	?	absent	absent	absent	absent to one
Alveoli	absent	absent (AD)	absent	?	?	absent	absent	absent
TL (mm)	56	(13–25) 15–20	29	17	120	31	24	20–45.5
TW (mm)	6	(1–1.5)	0.8	?	11	1.5	1.8	1.5–3.4
Opercular radii	55–160	15–25	32	32	62–136	18–23	25–55	21–33
Opercular tubercles on inner surface	present	?	absent (AD)	?	present	absent	present	absent
Constriction	well defined	well defined	well defined	well defined (AD)	absent	well defined	well defined	well defined
Sides of thoracic membrane	not fused	apron	apron	?	short apron	not fused	apron	short apron
Number of radioles per branchial lobe	10–35	6–15	9–10	13	43–61	16–30	22–30	12–37
Teeth of bayonet chaetae	2	2–4	not applicable	0	2	10	5	2
Proximal rasp of bayonet chaetae	absent	?	not applicable	present	absent	present	absent	absent

Regarding the *Serpula* species recorded in the Western Atlantic, *Serpula vossae* sp. n. differs from *Serpula vermicularis granulosa* Day, 1973, from Beaufort, North Carolina, because the former has a longer, deeper operculum, and lacks tubercles on the internal funnel surface (Figs 3A–D, 4H–J); while *Serpula vossae* sp. n. differs from *Serpula* sp. A (ten Hove and Wolf 1984) and *Serpula madrigalae* sp. n. because the former has a longer and deeper operculum, with more opercular radii (21–33) than the latter (*Serpula* sp. A has 18 radii, *Serpula madrigalae* sp. n. has 17).

*Serpula vossae* sp. n. differs from *Serpula* cf. *vermicularis*, recorded here from Nigeria, in the same characters mentioned for *Serpula vermicularis granulosa*, and, additionally in having fewer opercular radii in relation to the body length than the latter (Fig. 5).

#### Serpula cf. vermicularis

Figs 1E–F 2H–J 5 6

##### Material examined.

**Nigeria.** Five specimens (UMML 22.545), RV Pillsbury, sta. 248, Southeast of Lagos, 4°05’N, 5°40’E, 10-foot try-net, 33 m, May 13, 1965.

##### Description.

Tubes missing. Body light brown (preserved material only). TL= 18.5 mm (n=4, r:9.4–18.5, µ=36.6 ±10.3); THW= 1.7 mm (n=5, r:1.2–1.7, µ=1.6 ±0.2). Thoracic membranes and opercular peduncles of all the specimens damaged. Branchial crown with 27 radioles (n=4, r:19–27, µ=23.8 ±3.4) left, and 25 right (n=4, r:17–25, µ=22.5 ±3.7); lacking inter-radiolar membrane.

Peduncle smooth, with insertion on left (n=2) or right (n=2); lacking constriction between it and operculum, its position represented only by a slight change in color (Figs 1E, 2I). Club-shaped pseudoperculum present in all specimens.

Operculum with short, shallow symmetrical funnel; lacking bulbous basal part (Figs 1E, 2I). OL= 2 mm (n=4, r:1.3–2.1, µ=1.9 ±0.4), OD= 1.8 mm (n=4, r:1.1–1.8, µ=1.6 ±0.3). Interradial grooves 1/4 of funnel length (Fig. 2H). Funnel with up to 39 radii (n=4, r:33–39, µ=36.3 ±3.2) with blunt tips (Fig. 1E–F). Opercular inner surface lacking tubercles (Figs 1E–F, 2I).

Collar thick, with ventral and dorsal lobes short. Thorax consists of seven chaetigers. Collar chaetal fascicles symmetrical with regard to size and composition unlike in *Serpula vossae* sp. n. Bayonet chaetae with two blunt-elongate teeth, distal blade smooth, lacking proximal rasp (Fig. 2J); hooded (capillary) chaetae present.

Thoracic membranes apparently well developed (membranes damaged), narrowing toward posterior thorax, fused ventrally, forming a short apron. Remaining six thoracic chaetigers with hooded (limbate) chaetae of two sizes; saw-shaped uncini.

Abdomen with anterior achaetous region. Anterior and middle abdominal chaetigers with flat-trumpet chaetae. Posterior chaetigers with ‘capillary’ chaetae. Anterior and posterior uncini saw-shaped.

**Figure 6.**
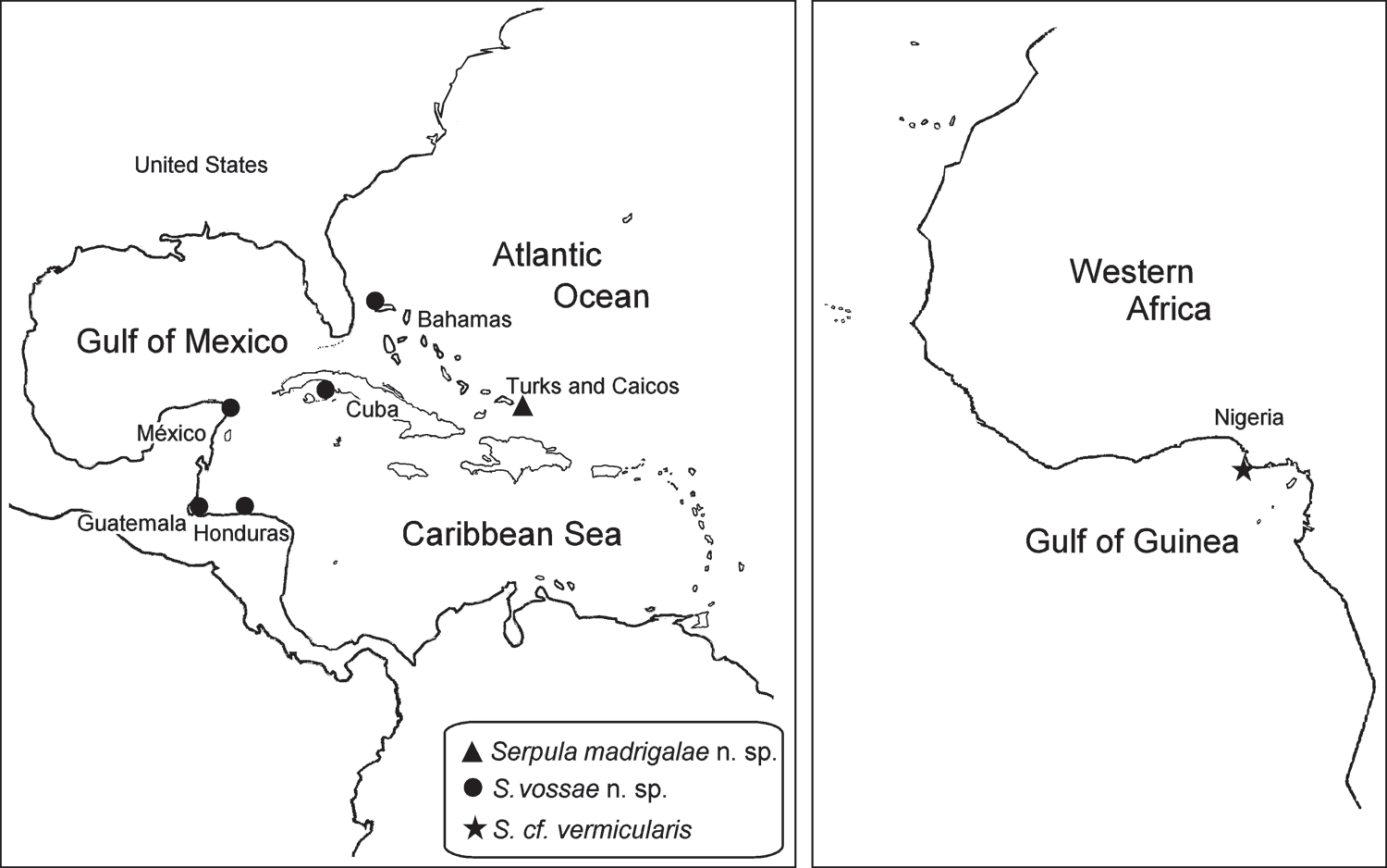
Distribution of *Serpula madrigalae* sp. n., *Serpula vossae* sp. n., and *Serpula* cf. *vermicularis*.

##### Variation.

Two specimens with a hyaline circle in radii tip (Fig. 1F). One specimen with few inconspicuous tubercles in interior funnel surface.

##### Distribution.

Nigeria, Gulf of Guinea (Fig. 6).

##### Ecology.

Sublittoral, 33 m.

##### Remarks.

*Serpula* cf. *vermicularis* resembles the nominal species; unfortunately, the tubes of all the specimens are missing. There are some differences with the nominal species, particularly with regard to the number of radii: *Serpula* cf. *vermicularis* has 33–39 opercular radii (Fig. 1F, 2I, 5), while Zibrowius (1968) recorded specimens from Marseille with more than 40 opercular radii, and Kupriyanova and Jirkov (1997) recorded specimens from Norway and Iceland with a mean of 50.8 opercular radii; and the proximal rasp of the bayonet chaetae: *Serpula* cf. *vermicularis* lacks a proximal rasp (Fig. 2J), while Rioja (1931) and Kupriyanova (1999, Table 1) mentioned that their specimens have a proximal rasp.

Zibrowius (1973) recorded several specimens as *Serpula vermicularis*, from Western Africa (from Angola to Morocco); unfortunately the description was too brief and did not included figures; however, Zibrowius (1973) mentioned that the specimens that he reviewed showed considerable variation.

### Genus *Spiraserpula* Regenhardt, 1961

**Type species. *Spiraserpula spiraserpula* Regenhardt, 1961** (fossil), by original designation.

#### Spiraserpula caribensis

Pillai & ten Hove, 1994

http://species-id.net/wiki/Spiraserpula_caribensis

Figs 7A–D 8

Spiraserpula caribensis Pillai & ten Hove 1994:68–76, Figs 3L, 14A–M, 15A–Y, 16A–K, Pls. 4E–F, 5A–E.

##### Type locality.

Awa Blancu, Curaçao.

##### Material examined.

**Panama Caribbean.** One specimen (ECOSUR P0615) Colon, Club Náutico, fouling prospection, June 3, 2002, S.I. Salazar-Vallejo leg. **Mexican Caribbean.** Nine specimens (ECOSUR P0614, P0616), two specimens (UMML 22.1061), two specimens (UMAR-Poly 110), Playa Azul, Cozumel, coral rock, 10 m, March 25, 2001, leg. H.A. ten Hove.

##### Description.

**Figure 7.**
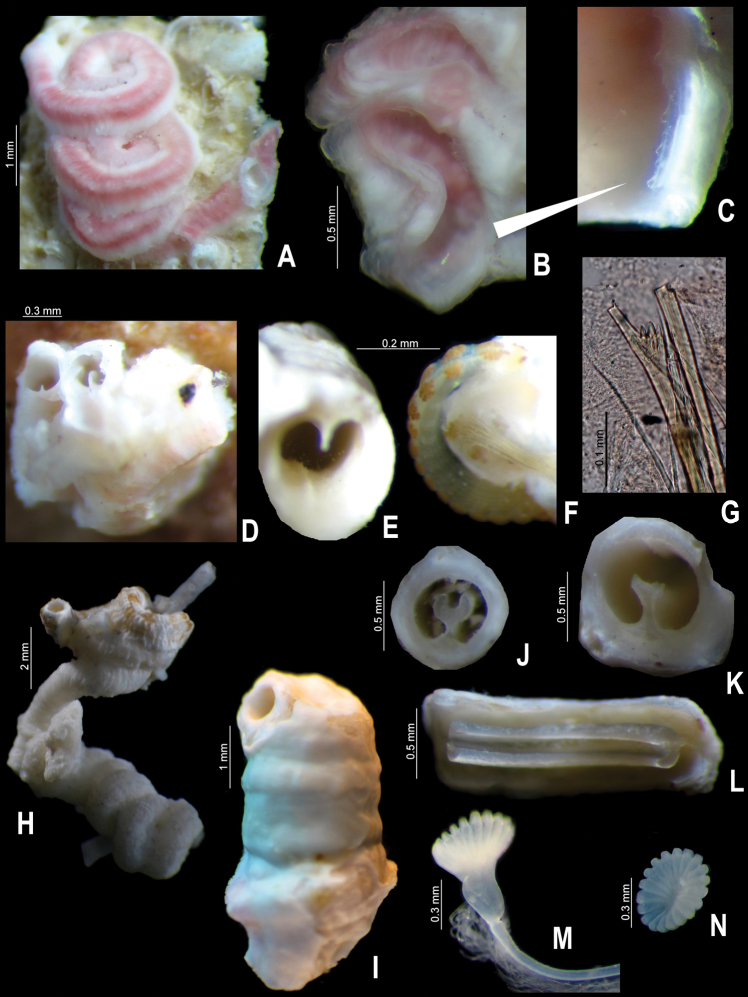
**A-D**: *Spiraserpula caribensis*, from Cozumel, UMAR-Poly 110 **A** complete tube **B–C** internal surface of the tube and detail of ventral internal ridge **D** other specimen with lateral internal ridges **E–F** *Spiraserpula karpatensis*, from Los Roques Islands, UMML 22.1055 **E** detail of the mouth tube **F** abdomen with gametes **G–L** *Spiraserpula ypsilon*, from Trinidad and Tobago, UMML 22.1059 **G** collar chaetae; from Bahamas, UMML 22.1056 **H** tube attached to *Pseudovermilia fuscostriata*; from Trinidad and Tobago, UMML 22.1059 **I** tube; from Honduras, UMML 22.1057 **J–K** tube in cross section **L** tube in longitudinal section **M–N** *Spiraserpula* sp., from Los Roques Islands, UMML 22.1060 **M–N** operculum, lateral and aboral views.

Some specimens forming tube aggregations; others were found isolated. Tubes sinuous or spiraled (Fig. 7A), with two internal ridges: mid-dorsal one smooth, mid-ventral one serrated (Fig. 7B–C), occasionally with two internal lateral ridges (Fig. 7D). Some tubes externally pinkish, others with two dorsal pink bands (Fig. 7A–B). Body brown to dark brown (preserved material only). The worms are damaged. Branchial crowns lost. Thorax with eight chaetigers, including collar fascicles. Abdomen damaged.

**Figure 8.**
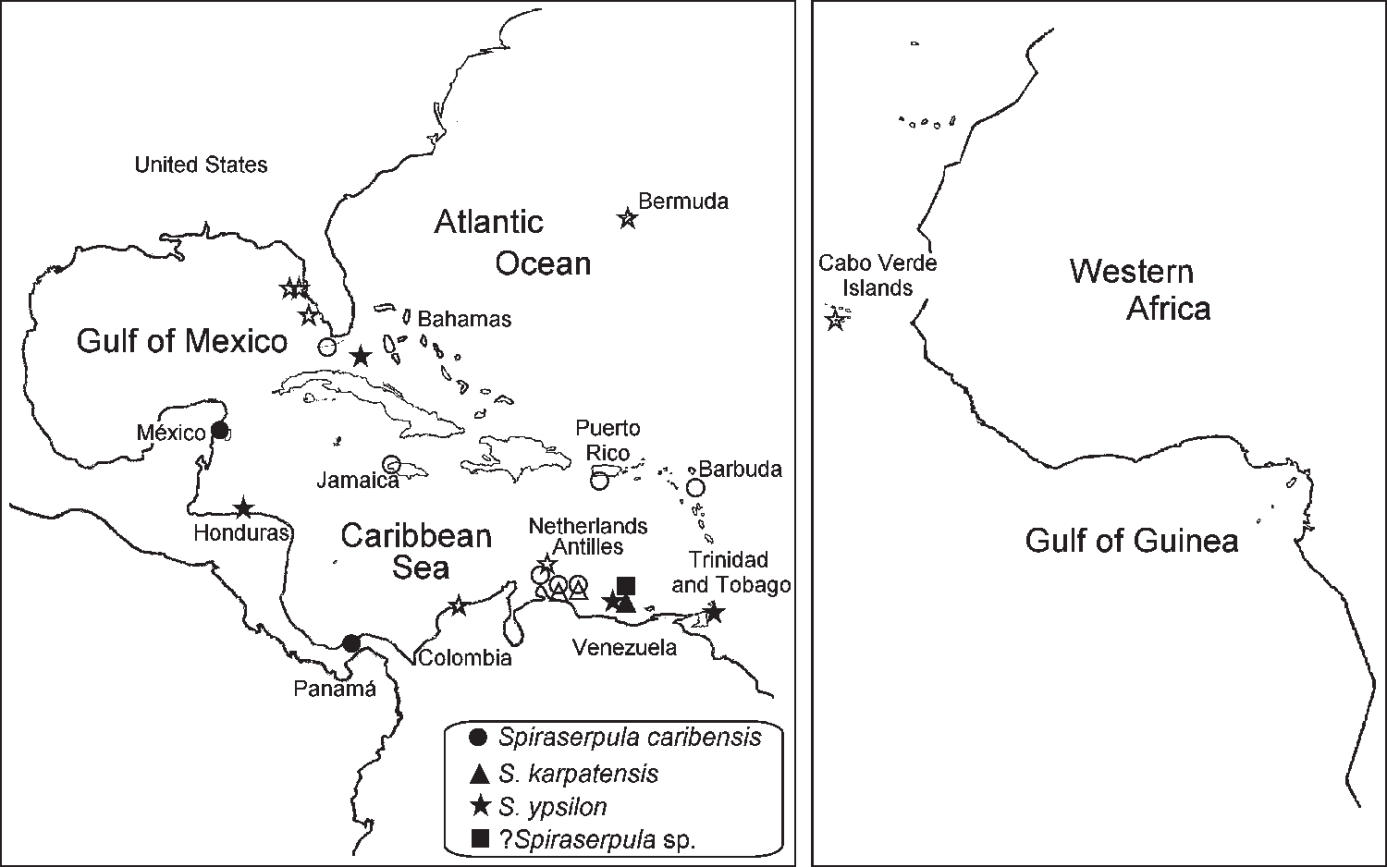
Distribution of *Spiraserpula caribensis*, *Spiraserpula karpatensis*, *Spiraserpula ypsilon* and ?*Spiraserpula* sp. Closed symbols denote examined material, open symbols literature records.

##### Distribution.

Caribbean, Florida and Pacific of Panama.

##### Ecology.

Intertidal to sublittoral, 10 m. On coral debris. Pillai and ten Hove (1994) recorded the species from 0–18 m deep.

##### Remarks.

*Spiraserpula caribensis*is easily distinguishable from the other Caribbean species by their pink tubes (Fig. 7A).

#### Spiraserpula karpatensis

Pillai & ten Hove, 1994

http://species-id.net/wiki/Spiraserpula_karpatensis

Figs 7E–F 8

Spiraserpula karpatensis Pillai & ten Hove 1994:64–65, Figs 3N, 11A–K.

##### Type locality.

Karpata, Bonaire.

##### Material examined.

**Venezuela.** One incomplete specimen and one empty tube (UMML 22.1055), RV Pillsbury, cruise 6806, sta. 745, North of Los Roques Islands, 11°58'N, 66°50'W, 10-feet otter trawl, 65 m, July 24, 1968.

##### Description.

Empty tube larger (Fig. 7E) than occupied one attached to empty tubes of *Spiraserpula ypsilon*. Tubes sinuous or spiraled, with two internal ridges: mid-dorsal one smooth, mid-ventral one serrated (Fig. 7E). Both tubes white, internal and externally (Fig. 7E). The branchial crown and thorax of incomplete specimen is missing. Abdomen partially transparent, with double packets of gametes in each segment (Fig. 7F).

##### Distribution.

Eastern Caribbean. Bonaire, Curaçao and Los Roques Islands.

##### Ecology.

Sublittoral, 65 m. On coral debris. Pillai and ten Hove (1994) recorded the species from depths of 10–30 m. The sample also contained two *Spiraserpula* species: *Spiraserpula ypsilon* and *Spiraserpula* sp., a chaetopterid tube, a lumbrinerid and several empty tubes of serpulids resembling *Protula* and *Vermiliopsis*.

##### Remarks.

*Spiraserpula karpatensis* resembles *Spiraserpula caribensis* with regard to the dorsal and ventral ridges (Fig. 7C, E); however, *Spiraserpula karpatensis* does not possess pinkish tubes unlike *Spiraserpula caribensis*.

#### Spiraserpula ypsilon

Pillai & ten Hove, 1994

http://species-id.net/wiki/Spiraserpula_ypsilon

Figs 7G–L 8

Spiraserpula ypsilon Pillai & ten Hove 1994:56–60, Figs 6A–K, 7A–T, 34G, Pl. 1B.

##### Type locality.

Brava, Cape Verde Islands.

##### Material examined.

**Bahamas.** Two empty tubes (UMML 22.1056), RV Gerda, cruise 6804, sta. 983, North of Elbow Cay, Bahamas, 24°05'N, 80°20'W, triangle dredge, 216 m, March 5, 1968). **Honduras.** One specimen (UMML 22.1057), RV Pillsbury, cruise 6802, sta. 629, Southwest of Honduras Cape, 15°58'N, 86°09'W, 41-feet otter trawl, 40 m, March 21, 1968. **Venezuela.** Two empty tubes (UMML 22.1058), RV Pillsbury, cruise 6806, sta. 745, North of Los Roques Islands, 11°58'N, 66°50'W, 10-feet otter trawl, 65 m, July 24, 1968. **Trinidad and Tobago.** One specimen (UMML 22.1059), one specimen (UMAR-Poly 111), RV Pillsbury, cruise 6907, sta. 840, East of Trinidad Island, 10°40'N, 60°37'W, 10-feet otter trawl, 33 m, sponges, July 1, 1969.

##### Description.

One specimen (UMML 22.1058) attached to tube of *Spiraserpula karpatensis* and another (UMML 22.1056) attached to *Pseudovermilia fuscostriata* tube (Fig. 7H). Tubes sinuous or strongly spiraled (Fig. 7H); in another the tube forms a very tight cylindrical spiral (Fig. 7I). Tubes with two internal longitudinal ridges: mid-dorsal one serrated, mid-ventral one Y-shaped (Fig. 7J–L); sometimes, along length of tube, Y-shaped ridge changes to smooth ridge. Tubes white (Fig. 7H–I). Body pale to dark brown (preserved material only). Worms damaged. Branchial crown with 5–6 radioles by branchial lobe. Collar damaged, lobes could not be observed. Bayonet chaetae with 3–4 blunt teeth (Fig. 7G); hooded (capillary) chaetae present. Thorax with seven chaetigers, including collar chaetae. Abdomen damaged.

##### Distribution.

Caribbean, Florida and Pacific of Panama.

##### Ecology.

Sublittoral, 33–216 m. On coral debris. Pillai and ten Hove (1994) recorded the species from 0.5 to 200 m. In the same samples studied there were other serpulids: *Spiraserpula karpatensis*, *Spiraserpula* sp., *Hydroides gairacensis*, *Hydroides* sp. 1, *Pomatostegus stellatus*, *Protula* sp., *Pseudovermilia fuscostriata*, *Pseudovermilia occidentalis*, *Salmacina huxleyi*, *Serpula vossae* sp. n., *Vermiliopsis annulata*, a chaetopterid tube, and other polychaetes: a lumbrinerid, several syllids, sipunculids and a vermetid shell.

##### Remarks.

*Spiraserpula ypsilon*is very similar to *Spiraserpula paraypsilon* Pillai & ten Hove, 1994, described from the Netherlands Antilles, mainly with regard to the internal ridges of the tube. However, some differences separate both species, mainly the absence of lateral tubercles in the thoracic uncini in *Spiraserpula ypsilon*, characteristic of *Spiraserpula paraypsilon*; additionally, *Spiraserpula ypsilon*has fewer radioles (6–7) than *Spiraserpula paraypsilon* (11).

#### ?Spiraserpula sp.

Figs 7M–N 8

##### Material examined.

**Venezuela.** One specimen (UMML 22.1060), RV Pillsbury, cruise 6806, sta. 745, North of Los Roques Islands, 11°58'N, 66°50'W, 10-feet otter trawl, 65 m, July 24, 1968.

##### Description.

Tube attached to a chaetopterid tube, is white and lacks any internal ridges characteristic of *Spiraserpula*. External surface with granular appearance, internally smooth. Body white, fragmented and damaged but complete. Branchial crown with nine radioles per lobe; lacking inter-radiolar membrane.

Peduncle smooth, inserted in right lobe, with well-defined constriction between it and operculum (Figs 7M). Pseudoperculum club-shaped. Operculum is zygomorphic; with a conspicuous bulbous basal part above constriction (Fig. 7M). Interradial grooves 1/3 of funnel length; 19 radii with rounded tips (Fig. 7N); inner surface smooth (Fig. 7N).

Collar damaged, lobes could not be observed. Bayonet chaetae with 2–3 sharp-elongate teeth; hooded (capillary) chaetae present. Thorax with eight chaetigers, including collar chaetae. Abdomen damaged, with approximately 61 segments, a distinct achaetous region absent between the thorax and abdomen.

##### Distribution.

Only recorded from Los Roques Islands, Venezuela.

##### Ecology.

Sublittoral, 65 m. The same sample contained other serpulids: *Spiraserpula karpatensis*, *Spiraserpula ypsilon*, several empty tubes of serpulids resembling *Protula* and *Vermiliopsis*, a chaetopterid tube, and a lumbrinerid.

##### Remarks.

Most of the tube belonging to this specimen is missing and the remaining fragments lacked the internal ridges characteristic of *Spiraserpula*. The operculum of this *Spiraserpula* sp. resembles that of *Spiraserpula karpatensis*, *Spiraserpula plaiae* Pillai & ten Hove, 1994 and *Spiraserpula sumbensis* Pillai & ten Hove, 1994; the former two are from Caribbean and the latter is from Indonesia. Due to the loss of the rest of the tube the present specimen cannot be assigned to species. It may be a juvenile stage of another genus, such as *Crucigera* or *Serpula*.

## Discussion

Despite having reviewed 158 lots of serpulids from the same number of stations collected during past deep sea expeditions, specimens of *Serpula* were found only at six stations (3.8%), which combined with the fact that there were only two previous records of *Serpula* (Day 1973, ten Hove and Wolf 1984) from the U.S. Atlantic, indicates that the genus is very rare in the Western Atlantic.

However, the original descriptions of the species mentioned in the remarks, indicate that many are incomplete, unclear or contradictory with respect to the figures provided. Descriptions need to be standardized and include as many characters as possible, as argued extensively by ten Hove and Jansen-Jacobs (1984), Kupriyanova (1999), and ten Hove and Kupriyanova (2009).

As regards *Spiraserpula*, another little-known serpulid genus in the Caribbean closely similar to *Serpula*, complete descriptions of species were made in an important and recent revision of the genus, including most species from the Caribbean (Pillai and ten Hove 1994). Unfortunately, due to the characteristics of the internal tube structures and small size of the specimens, their manipulation and study of *Spiraserpula* is more difficult as compared to other serpulids.

## Supplementary Material

XML Treatment for Serpula madrigalae

XML Treatment for Serpula vossae

XML Treatment for Serpula cf. vermicularis

XML Treatment for Spiraserpula caribensis

XML Treatment for Spiraserpula karpatensis

XML Treatment for Spiraserpula ypsilon

XML Treatment for ?Spiraserpula sp.
